# Roles of the quantification of serine in the brain

**DOI:** 10.17179/excli2023-6857

**Published:** 2024-01-05

**Authors:** Jia-Meng Li, Ya-Zhi Bai, Shuang-Qing Zhang

**Affiliations:** 1 Department of Nutrition and Metabolism, National Institute for Nutrition and Health, Chinese Center for Disease Control and Prevention, 27 Nanwei Road, Beijing 100050, China

## ⁯

As a nonessential amino acid, serine has two enantiomers of D-serine and L-serine, in which *in vivo* L-serine is derived from dietary intake, generation of glycine and one-carbon units, breakdown of proteins and phospholipids, *de novo* synthesis from glucose and conversion from D-serine, and D-serine is produced from glycine and L-serine (Bai et al., 2023[1]). Serine is essential for the healthy development and function of the brain. In the brain, L-serine is mainly synthesized from glucose due to the low blood-brain barrier permeability of L-serine and glycine, and is considered a conditionally essential amino acid for the brain. D-serine is a potent neurotransmitter and a gliotransmitter for the regulation of the activities of neurons in the brain (Li and Zhang, 2023[3]). Therefore, the quantification of D-serine and L-serine is very important for the brain. 

For the quantification of D-serine and L-serine, analytical methods using high performance liquid chromatography (HPLC) or capillary electrophoresis (CE) coupled to various detection systems, such as ultraviolet-visible (UV) detector, fluorescence detector, electrochemical detector (ECD) and mass spectrometry (MS) detector, have been established (Le Douce et al., 2020[2]; Lorenzo et al., 2013[4]; Shikanai et al., 2022[5]). An easy-to-use UV detection is less selective and sensitive, and MS provides high selectivity and sensitivity. With UV and fluorescence detection, pre- or post-column derivatization is often applied due to the lack of a chromophore of serine. Fluorometric detection combined with fluorescence derivatization is often used due to its simplicity, convenience and sensitivity. Usually, it is necessary to employ chiral stationary phase or diastereomer derivatization reagent for the simultaneous separation and determination of D-serine and L-serine. Typical derivatization reagents include ortho-phthalaldehyde (OPA), N-acetyl-cysteine (NAC), 4-fluoro-7-nitro-2,1,3-benzoxadiazole (NBD-F), naphthalene-2,3-dialdehyde, dimethylaminonaphthalene-1-sulfonyl chloride, fluorescamine, 9-fluorenylmethylchloroformate, fluorescein-5-isothiocyanate, and 3-(4-carboxybenzoyl)-2-quinolinecarboxaldehyde. Derivatization reactions are laborious and time-consuming, as well as sometimes lack reproducibility.

Prior to the analysis of serine in biological matrix including brain tissue, biological samples were usually subject to homogenization, centrifugation, extraction and derivatization. For rat brain sample, HPLC-ECD was used to quantify D-serine and L-serine derivatized with OPA and NAC on a C18 column with a mobile phase of phosphate buffer and methanol containing ethylenediaminetetraacetic acid sodium (Shikanai et al., 2022[5]). CE coupled to laser-induced fluorescence detection was employed to measure D-serine and L-serine in urine and hippocampus using NBD-F as a derivatizing agent (Lorenzo et al., 2013[4]). Enantioselective chromatographic separation of D-serine and L-serine in mouse brain was achieved on a chiral crown ether column CROWNPAK CR-I (+) with an isocratic mobile phase of 0.3 % trifluoroacetic acid in 10 % acetonitrile by HPLC-MS/MS without a time-consuming and poor qualitative derivatization procedure (Le Douce et al., 2020[2]). The analytical results of serine disclosed that the impairment of glycolysis-derived L-serine production in astrocytes contributed to cognitive deficits via D-serine in Alzheimer's disease.

In conclusion, a fast, simple and sensitive method without derivatization needs to be established for the simultaneous quantification of D-serine and L-serine to support cognition investigation.

## Notes

Jia-Meng Li and Ya-Zhi Bai contributed equally as first author.

## Declaration

### Conflict of interest

The authors declare no conflict of interest.

### Acknowledgments

None.

### Fundings

The authors declare that no funds, grants, or other support were received during the preparation of this manuscript.

## References

[1] Bai YZ, Li JM, Zhang SQ (2023). Potential novel mechanism of selenium on cognition. Metab Brain Dis.

[2] Le Douce J, Maugard M, Veran J, Matos M, Jego P, Vigneron PA (2020). Impairment of glycolysis-derived L-Serine production in astrocytes contributes to cognitive deficits in Alzheimer's Disease. Cell Metab.

[3] Li JM, Zhang SQ (2023). Roles of serine in neurodegenerative diseases. EXCLI J.

[4] Lorenzo MP, Navarrete A, Balderas C, Garcia A (2013). Optimization and validation of a CE-LIF method for amino acid determination in biological samples. J Pharm Biomed Anal.

[5] Shikanai H, Ikimura K, Miura M, Shindo T, Watarai A, Izumi T (2022). Separation and detection of D-/L-serine by conventional HPLC. MethodsX.

